# Acute stress induces an inflammation dominated by innate immunity represented by neutrophils in mice

**DOI:** 10.3389/fimmu.2022.1014296

**Published:** 2022-09-29

**Authors:** Lanjing Tang, Nannan Cai, Yao Zhou, Yi Liu, Jingxia Hu, Yalin Li, Shuying Yi, Wengang Song, Li Kang, Hao He

**Affiliations:** ^1^ Department of Immunology, Shandong First Medical University & Shandong Academy of Medical Sciences, Jinan, China; ^2^ Shandong Provincial Key Laboratory for Rheumatic Disease and Translational Medicine, The First Affiliated Hospital of Shandong First Medical University & Shandong Provincial Qianfoshan Hospital, Jinan, China; ^3^ Department of Ophthalmology, Taian Maternity and Child Health Hospital, Taian, China; ^4^ Department of Pediatrics, Taian Maternity and Child Health Hospital, Taian, China

**Keywords:** acute stress, bioinformatics, inflammation, neutrophils, peripheral blood

## Abstract

It is well known that psychological stress could affect the immune system and then regulate the disease process. Previous studies mostly focused on the effects of chronic stress on diseases and immune cells. How acute stress affects the immune system remains poorly understood. In this study, after 6 hours of restraint stress or no stress, RNA was extracted from mouse peripheral blood followed by sequencing. Through bioinformatics analysis, we found that when compared with the control group, differentially expressed genes in the stress group mainly displayed up-regulated expression. Gene set enrichment analysis results showed that the enriched gene terms were mainly related to inflammatory response, defense response, wounding response, wound healing, complement activation and pro-inflammatory cytokine production. In terms of cell activation, differentiation and chemotaxis, the enriched gene terms were related to a variety of immune cells, among which neutrophils seemed more active in stress response. The results of gene set variation analysis showed that under acute stress, the inflammatory reaction dominated by innate immunity was forming. Additionally, the concentration of serum IL-1β and IL-6 increased significantly after acute stress, indicating that the body was in an inflammatory state. Importantly, we found that acute stress led to a significant increase in the number of neutrophils in peripheral blood, while the number of T cells and B cells decreased significantly through flow cytometric analysis. Through protein-protein interaction network analysis, we screened 10 hub genes, which mainly related to inflammation and neutrophils. We also found acute stress led to an up-regulation of *Ccr1, Ccr2, Xcr1* and *Cxcr2* genes, which were involved in cell migration and chemotaxis. Our data suggested that immune cells were ready to infiltrate into tissues in emergency through blood vessels under acute stress. This hypothesis was supported in LPS-induced acute inflammatory models. After 48 hours of LPS treatment, flow cytometric analysis showed that the lungs of mice with acute stress were characterized by increased neutrophil infiltration, decreased T cell and B cell infiltration. Immunohistochemical analysis also showed that acute stress led to more severe lung inflammation. If mice received repeat acute stress and LPS stimulation, the survival rate was significantly lower than that of mice only stimulated by LPS. Altogether, acute stress led to rapid mobilization of the immune system, and the body presented an inflammatory state dominated by innate immune response represented by neutrophils.

## Introduction

Psychological stress is the process of psychological and physiological changes caused by the body’s awareness of the threat of stressors through cognition and evaluation (1, 2). Psychological stress beyond individual tolerance is often the source of many diseases. Depression, cardiovascular diseases, tumors, inflammatory bowel diseases and autoimmune diseases are closely related to psychological stress (3–8). With the transformation from traditional medical model to biopsychosocial model (9), the role of psychological stress in the occurrence and development of diseases has drawn increasing attention.

Stress is usually be regarded as the experience of anticipating or encountering adversity, while stress response is the non-specific response of the body to stressors. According to the duration of stimulation, stress can be divided into acute stress and chronic stress (10). The effects of acute stress and chronic stress are different. It is generally believed that chronic stress is harmful to health (11–13), while acute stress is conducive to life survival (14). Current evidence supports that stress regulates the process of disease by affecting the immune system (15–17). At present, most studies focused on the effects of chronic stress on diseases and immune cells. However, acute stress is the basis of chronic stress and may determine the direction of chronic stress-induced response. How acute stress affects the immune system remains poorly understood. Here, we studied the effects of acute stress on gene transcription in peripheral blood cells of mice through bioinformatics analysis, and detected the changes of blood cell populations under acute stress. We further explored the effect of acute stress on the pathological state of the body. Given that the effect of acute stress gradually attenuates with the removal of stressor, it is a good choice to verify in the acute inflammation model. Therefore, LPS-induced acute inflammation model widely used in medical research was selected as the verifier. Our data showed that acute stress led to rapid mobilization of the immune system, and the body presented an inflammatory state dominated by innate immune response represented by neutrophils.

## Materials and methods

### Mice

Female C57BL/6 (B6; H-2 Kb) mice, aged 6-8 weeks, were purchased from Charles River (Beijing, China). All mice were maintained in specific pathogen–free (SPF) conditions. All protocols involving animals were in compliance with the experimental guidelines approved by the Laboratory Animal Care Committee of Shandong First Medical University & Shandong Academy of Medical Sciences.

### Stress model and LPS administration

Stress model was prepared as described previously (18). Briefly, mice were placed in a 50-ml conical centrifuge tube filled with multiple punctures to allow ventilation without food and water supply. The control littermates were kept in normal cage and were not supplied with food and water during the stress. After 6 hours of restrain stress, peripheral blood cells were obtained for flow cytometric analysis. In other experiments, mice received restrain stress followed by intraperitoneal injection with 2 mg/kg LPS diluted in PBS (E.coli, serotype 0111:b4; Sigma-Aldrich) or the same volume of PBS. 48 hours later, mice were killed and the lung tissues were taken for flow cytometric analysis and H&E staining. In other cases, mice received daily restraint stress and LPS injection, and their mortality was monitored.

### ELISA assay

After mice received restraint stress or no stress for 6 hours, mouse serum was collected. Concentration of IL-1β, IL-6 and TNF-α in the serum were determined by ELISA Ready-SET-Go Kit (eBioscience) according to the manufacturers’ protocol.

### Bulk RNA-seq

Peripheral blood was obtained from mice with or without stress. RNA were extracted by using Illumina TruSeq RNA Sample Prep Kit (Illumina) according to the manufacturer’s instructions. RNA-seq libraries were prepared using standard Illumina protocols, followed by sequencing on an Illumina NovaSeq6000 instrument. RNA sequencing data have been saved in the NCBI GEO database for public access. GEO accession number is GSE210252.

### DEG identification

Dimension reduction analysis of RNA-seq was performed using R package Rtsne (19). R package DESeq2 was used to identify differentially expressed genes (DEG). Log2 fold change (FC) was used to evaluate the degree of gene expression difference. The adjusted p value (adj.P.Val) was used to avoid the occurrence of false-positive results. Compared with control group, genes with | log2FC | >1 and adj.P.Val < 0.01 were regarded as DEG in stress group. R package ggplot2 and pheatmap were used to visualize the identified DEG by generating volcano plot and heat maps respectively.

### PPI construction and hub gene identification

Based on the selected DEG, we used an online tool for searching of interacting genes (string; http://string.embl.de/) to predict the functional interactions between proteins (20). Based on the STRING database, a protein-protein interaction (PPI) network was constructed by using genes with confidence score ≥ 0.4. Subsequently, the network data were input into Cytoscape (v3.7.2) software. The Molecular Complex Detection (MCODE) was performed to screen modules of PPI network (degree cutoff=2, node cutoff=0.2, k-core=2, max.depth=100). Ten hub genes were identified by maximum clique centrality (MCC) algorithm.

### Functional analyses

Gene set enrichment analysis (GSEA) and gene set variation analysis (GSVA) were used for functional analyses (21, 22). Briefly, GSEA was performed on the whole transcriptome by using R package clusterprofiler (23). Gene Ontology (GO) enrichment analysis included cellular component (CC) analysis, molecular function (MF) analysis and biological process (BP) analysis. GOplot packages of R was used to visualize the enriched gene terms (24). “GO BP” gene terms were downloaded from the molecular signature database, and GSVA was performed by using R package GSVA to reveal the functional changes between the stress group and the control group. For GSEA and GSVA, adj.P.Value < 0.05 was considered statistically significant.

### Preparation of single cell suspensions

Single cell suspensions were prepared as described previously (25). Briefly, blood leukocytes were purified by lysing erythrocytes with ACK Lysing buffer. Lung parenchyma was collected from mice and digested with collagenase IV (1mg per ml) for 1h at 37°C followed by resuspension in 30% Percoll (GE Healthcare, Uppsala, Sweden)for centrifugation at 1200g for 20 min at room temperature. Then cells were incubated with ACK Lysing buffer to remove erythrocytes.

### Flow cytometry

Single cell suspensions were first blocked with anti-Fcr III/II receptor mAb (2.4G2) followed by staining with fluorescence-conjugated mAb for CD45 (30-F11), CD19 (1D3), CD3 (145-2C11), CD11b (M1/70), Ly6G (1A8), Ly6C (HK1.4), NK1.1 (PK136). All mAbs were obtained from Thermo Fisher (Thermo Fisher Scientific Inc., Waltham, MA, USA). CD45^+^ cells were gated to analyze the CD3^-^CD19^+^ B cells, CD3^+^NK1.1^-^ T cells and CD3^-^NK1.1^-^ NK cells. CD45^+^CD11b^+^ cells were gated to analyze Ly6C^+^Ly6G^high^ neutrophils and Ly6C^high^Ly6G^-^ monocytes. Flow cytometry gating strategy was shown in **Supplementary Figure 1**. For cell count, stained cells were collected at high speed for 50 seconds and counted by flow cytometry using the Aria II Flow Cytometer (BD Bioscience).

### Statistical analysis

Statistical significance of differences was determined by Student’s t tests (2 groups) or ANOVA (at least 3 groups). Data were presented as mean ± SD, and P < 0.05 was considered statistically significant. GraphPad Prism 5 software (Graphpad, software, Inc, LaJolla, CA, USA) was used for statistical analysis.

## Results

### Acute stress changes the gene expression profile of peripheral blood cells

**Figure 1:**
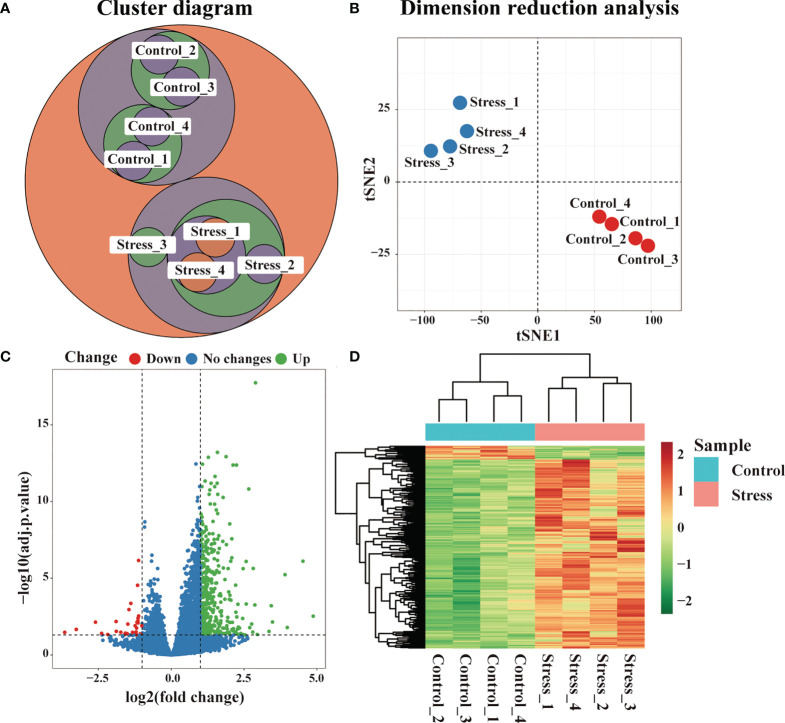
Changes of gene expression profile in peripheral blood of mice under acute stress. After 6 hours of restraint stress (n=4) or no stress (n=4), RNA of peripheral blood was extracted and sequenced, and DEG were further screened. **(A)** Cluster analysis of all samples. **(B)** Dimension reduction analysis of all samples by t-SNE method. **(C)** Difference of gene expression between the stress group and the control group displayed by volcano graph. **(D)** Difference of gene expression between the stress group and the control group displayed by heatmap graph.

RNA sequencing was performed on the peripheral blood of 6-hour stressed mice and control mice, and the samples were clustered according to the gene expression profile. As shown in **Figure 1A**, 8 samples could be classified into two groups, which was completely consistent with the experimental design. Dimension reduction analysis of RNA-seq was also performed using t-SNE method. We constructed a low dimensional embedding of high-dimensional gene expression data, and obtained two-dimensional analogues of clusters. As shown in **Figure 1B**, these two clusters just responded to the stress group and the control group. Our data indicated that the quality control of RNA-seq was good. Gene differential expression analysis was subsequently carried out on the two groups of samples. Taking | log2FC | >1 & adj.P.Val < 0.01 as the threshold, our data showed that there were significant differences in the gene expression profiles between the stress group and the control group. Compared with the control group, 307 genes displayed up-regulated expression and 12 genes displayed down-regulated expression in the stress group (**Figure 1C, D**). All the DEG were listed in **Supplementary data**.

### Acute stress affect the gene expression at immune response level

**Figure 2:**
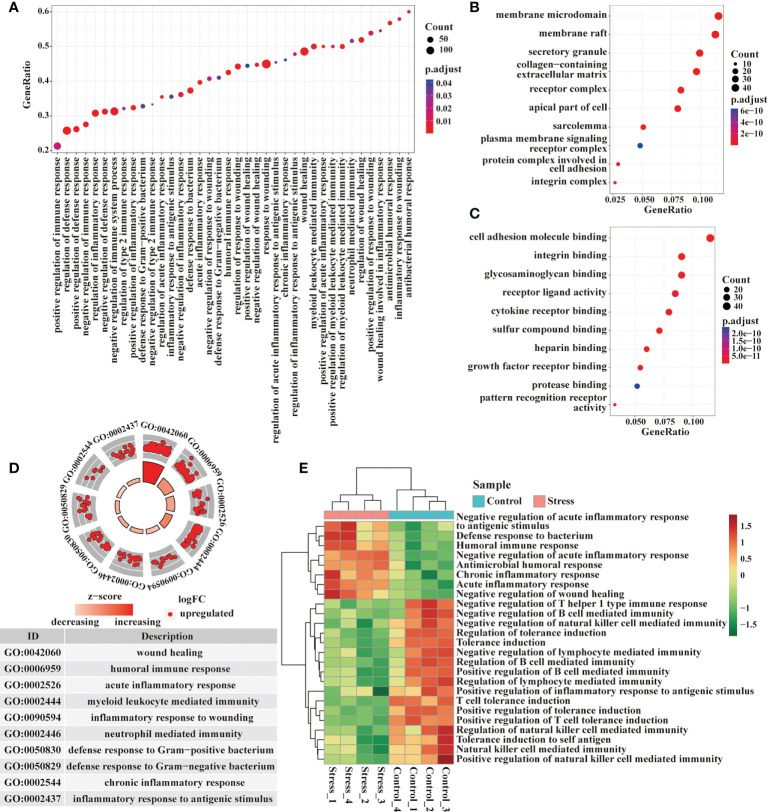
Analysis of gene expression at immune response level by GSEA and GSVA. **(A)** GO enrichment analysis of the whole transcriptome was performed by GSEA, and the biological processes represented by the gene terms were visualized at the immune response level. **(B, C)** Genes from these gene terms were selected for another GO enrichment analysis, and the top 10 CC and MF were visualized. **(D)** Select some important gene terms related to immune response, and then visualize the gene expression. **(E)** Meanwhile, GSVA was performed on RNA-seq, and the differentially expressed gene terms were visualized at the immune response level.

Through the GO enrichment analysis of the whole transcriptome by GSEA, there enriched many gene terms under acute stress. At the level of immune response, the biological processes represented by gene terms mainly involved inflammatory response to wounding, wound healing, defense response to bacterium, acute inflammatory response, chronic inflammatory response, humoral immune response, regulation of inflammation and immune response (**Figure 2A**). The molecules corresponding to these gene terms were mainly distributed in cell membrane, secretory granule, receptor complex, integrin complex and so on (**Figure 2B**), and their functions were mainly related to cell adhesion, integrin binding, glycosaminoglycan binding, receptor ligand activity, cytokine receptor binding, pattern recognition receptor activity, etc. (**Figure 2C**). We selected some important gene terms related to immune response and found that all genes in these gene terms showed up-regulated expression (**Figure 2D**). The results of GSVA showed that when compared with control group, some gene terms such as defense response to bacterium, acute/chronic inflammatory response displayed up-regulated expression, while other gene terms such as tolerance induction displayed down-regulated expression in the stress group (**Figure 2E**). Our data suggested that acute stress might trigger inflammatory response to cope with the upcoming unknown threat.

### Acute stress affect the gene expression at immune molecular level

**Figure 3:**
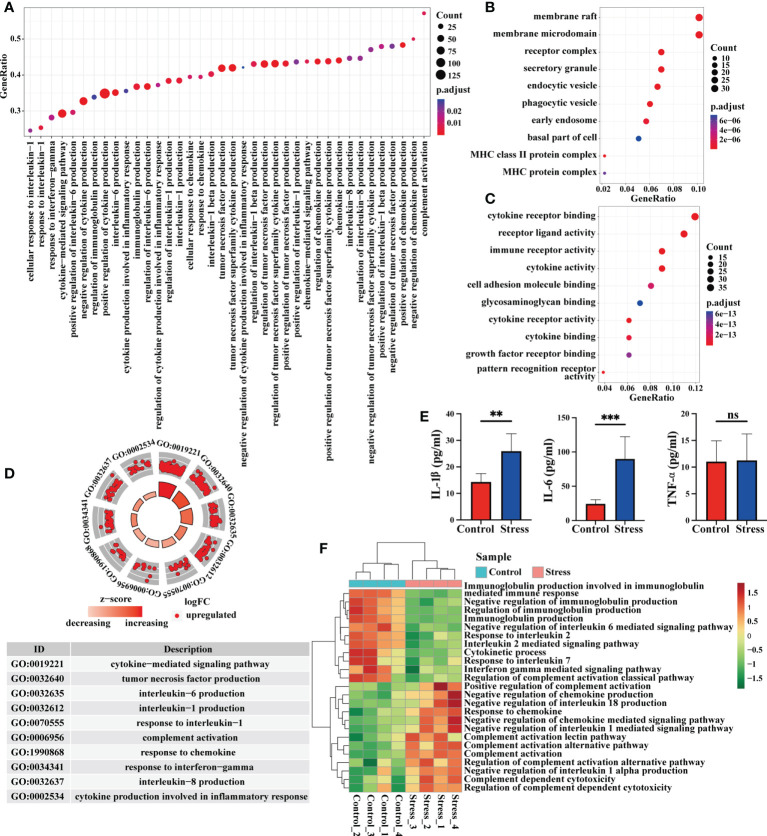
Analysis of gene expression at immune molecular level by GSEA and GSVA. **(A)** GO enrichment analysis of the whole transcriptome was performed by GSEA, and the biological processes represented by the gene terms were visualized at the immune molecular level. **(B, C)** Genes from these gene terms were selected for another GO enrichment analysis, and the top 10 CC and MF were visualized. **(D)** Select some important gene terms related to immune molecules, and then visualize the gene expression. **(E)** Concentration of serum proinflammatory cytokines IL-6, TNF-α and IL-1β were detected by ELISA in mice with or without stress (n=5, per group). **P < 0.01, ***P < 0.001. Means ± SD are shown. Data shown are representative of 2 independent experiments. **(F)** Meanwhile, GSVA was performed on RNA-seq, and the differentially expressed gene terms were visualized at the immune molecular level.

Immune molecules are usually used by immunocytes to interact with each other and exert effects, so we analyzed the enriched gene terms by GSEA at the immune molecular level. The biological processes represented by the enriched gene terms mainly involved cytokine-mediated signal pathway, production of IL-1, IL-6, IL-8, TNF, responses to IL-1, IFN-γ and chemokines, as well as complement activation (**Figure 3A**). The molecules corresponding to these gene terms were mainly distributed in cell membrane, receptor complex, secretory granule, endocytic vesicle, phagocytic vesicle and so on (**Figure 3B**), and their functions were mainly related to the cytokine receptor binding, immune receptor activity, cytokine activity, cell adhesion, glycosaminoglycan binding, cytokine receptor activity, cytokine binding, pattern recognition receptor activation, etc. (**Figure 3C**). In some important gene terms related to immune molecules, all genes showed up-regulated expression (**Figure 3D**), suggesting that acute stress might trigger the mobilization of immune molecules. We further detected the serum proinflammatory cytokines in mice by ELISA. Our data showed that IL-1β and IL-6 increased significantly in the circulation after acute stress, but TNF-α did not change significantly. The promoting effect of acute stress on serum IL-6 was much stronger than that on IL-1β (**Figure 3E**). The results of GSVA showed that when compared with control group, some gene terms such as complement activation and response to chemokine displayed up-regulated expression, while other gene terms such as immunoglobulin production, response to IL-2 and IL-7 displayed down-regulated expression in the stress group (**Figure 3F**).

### Acute stress affect the gene expression at the cellular level and the number of blood cells

**Figure 4:**
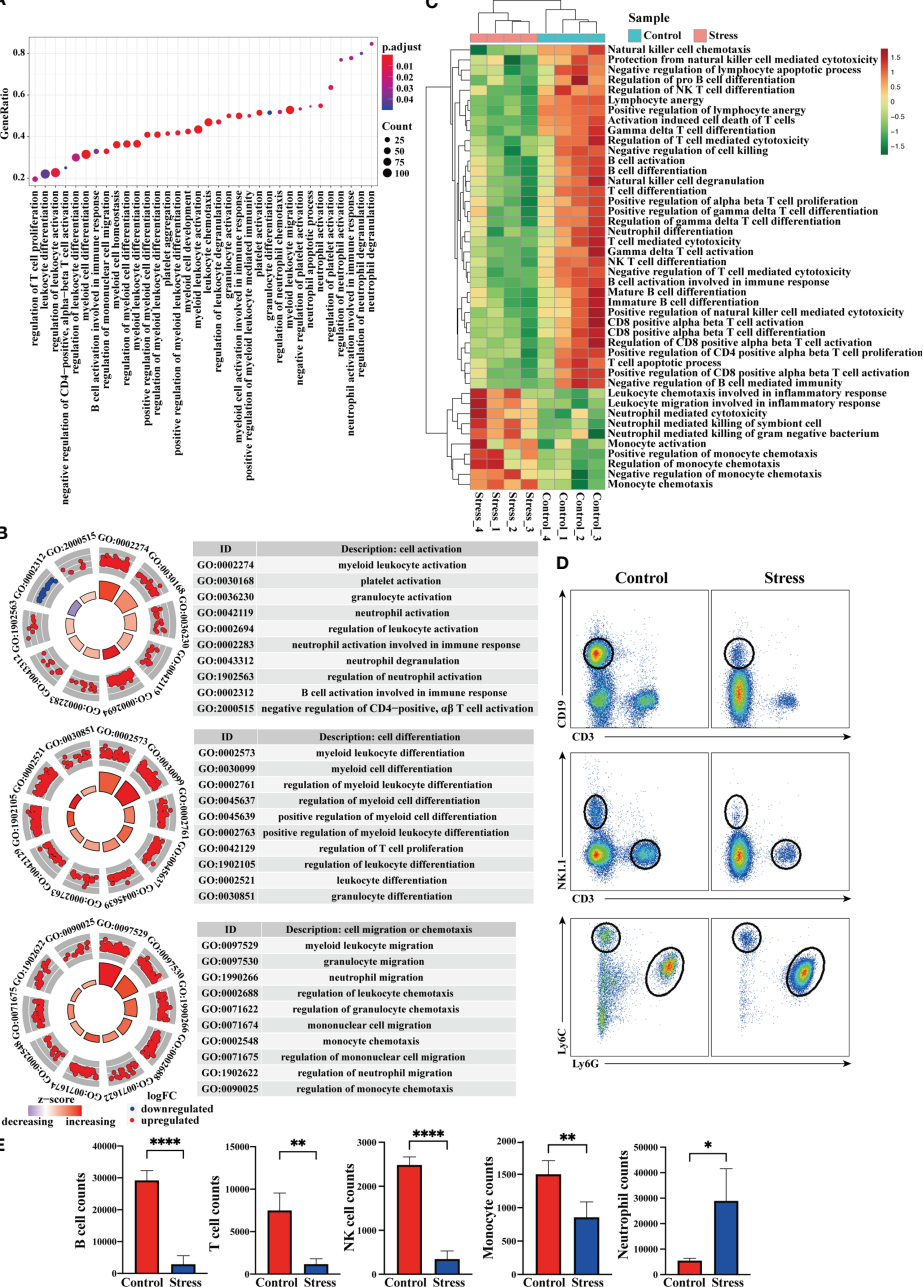
Analysis of gene expression at the cellular level and cell number in peripheral blood. **(A)** GO enrichment analysis of the whole transcriptome was performed through GSEA, and the biological processes represented by enriched gene terms were visualized at the cellular level. **(B)** Select some important gene terms related to cell activation, differentiation and migration, and then visualize the gene expression. **(C)** GSVA was performed on RNA-seq, and the differentially expressed gene terms were visualized at the cellular level. **(D)** At the same time, flow cytometry was used to analyze the changes of immunocytes in the peripheral blood between stress group and control group (n=4, per group). **(E)** Histograms represent the cell number counted by flow cytometry. Data are representative of 3 independent experiments. *P < 0.05, **P < 0.01, ****P < 0.0001. Means ± SD are shown.

Immune cells are the core components that reflect the immune function of the body, we thus analyzed the enriched gene terms by GSEA at the cellular level (**Figure 4A**). In terms of cell activation, the biological processes represented by the enriched gene terms mainly involved granulocyte activation, neutrophil activation, neutrophil degranulation, B cell activation, CD4^+^ αβT cell activation, platelet activation and the regulation of cell activation. In terms of cell development and differentiation, the biological processes represented by the enriched gene terms mainly involved leukocyte differentiation, granulocyte differentiation, regulation of T cell proliferation and regulation of cell differentiation. In terms of cell chemotaxis or migration, the biological processes represented by the enriched gene terms mainly involved granulocyte migration, neutrophil migration, monocyte chemotaxis, mononuclear cell migration and regulation of cell chemotaxis or migration (**Figure 4B**). It was worth noting that genes in the gene terms related to B cell activation and T cell activation showed down-regulated expression (**Figure 4B**). The results of GSVA showed that when compared with control group, some gene terms such as neutrophil mediated killing, monocyte activation and chemotaxis displayed up-regulated expression, while other gene terms such as B cell activation and differentiation, T cell activation, differentiation and function, NK cell degranulation, NKT cell differentiation displayed down-regulated expression in the stress group (**Figure 4C**). Flow cytometric analysis showed that acute stress led to a sharp reduction of T cells, B cells, NK cells and monocytes as well as significant increment of neutrophils in peripheral blood (**Figure 4D, E**).

### Identifying the hub genes and analyzing migration-related genes from DEG

**Figure 5:**
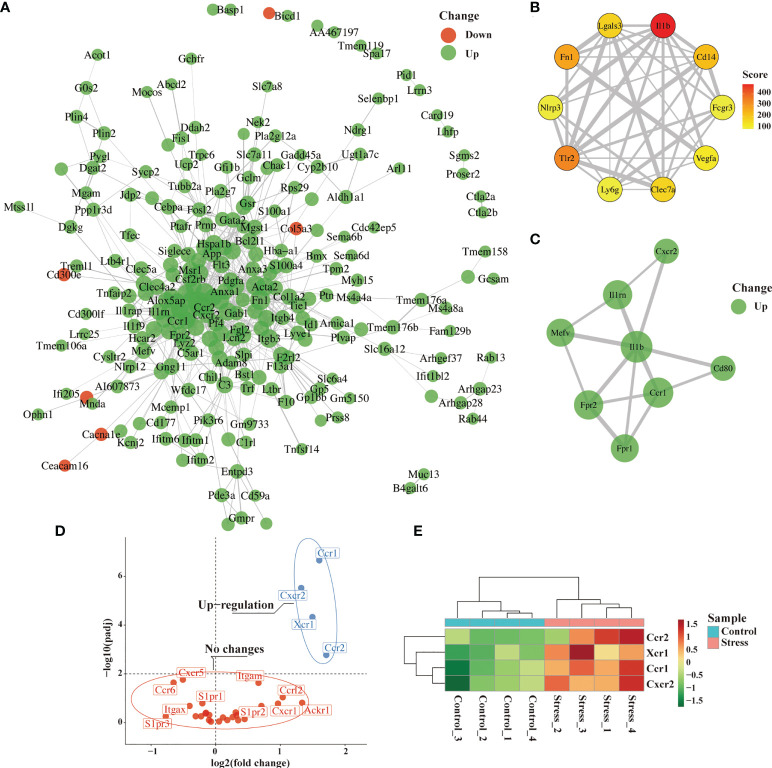
Identification of hub genes and analysis of migration-related genes from DEG. After 6 hours of restraint stress or no stress (n=4, per group), RNA extracted from peripheral blood was sequenced and analyzed. **(A)** The selected DEG were used for PPI network analysis online. **(B, C)** Hub gene and modules were screened from PPI network through Cytoscape software. **(D, E)** Genes related to cell migration or chemotaxis were analyzed and visualized by volcano graph and heatmap graph.

**Figure 6:**
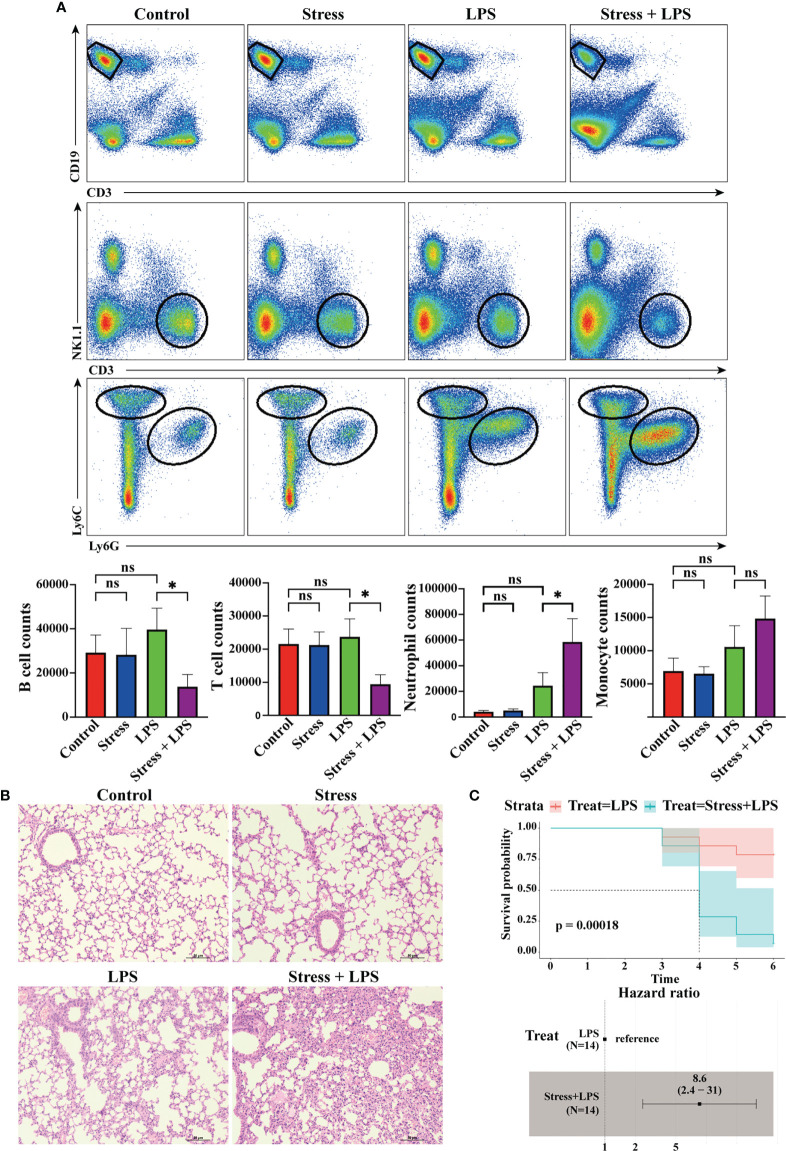
Acute stress caused excessive lung inflammation in LPS-treated mice. Mice were intraperitoneally injected with LPS after 6 hours of restraint stress or no stress. **(A)** 48 hours later, the inflammatory cells infiltrated in the lungs were detected by flow cytometry (n=4, per group). Histograms represent the cell number counted by flow cytometry. **(B)** Meanwhile, lungs were sectioned and stained with H&E to observe the pathological changes. **(C)** Mice received LPS stimulation with or without restraint stress every day, and the mortality and hazard ratio were calculated (n=4, per group). *P < 0.05, Means ± SD are shown. Data are representative of 3 independent experiments. ns, no significance.

To further predict the interaction network among the molecules corresponding to the DEG, we performed PPI network analysis online and visualized it using R package ggraph. As predicted, PPI network was full of complex molecular communication (**Figure 5A**). We next imported the PPI network data into Cytoscape software and identified 10 major hub genes containing *Il1b*, *Tlr2*, *Fn1*, *Cd14*, *Lgals3*, *Clec7a*, *Vegfa*, *Nlrp3*, *Ly6g and Fcgr3* (**Figure 5B**). *Il1b* got the highest score among hub genes, and the cytokine encoded by it is crucial for the occurrence of inflammation. We further used the MCODE plug-in to calculate and screen the co-expression module containing *Il1b*. As shown in **Figure 6C**, there were 8 up-regulated genes in the module including *Il1b*, *Il1rn*, *Ccr1*, *Cxcr2*, *Mefv*, *Cd80*, *Fpr1* and *Fpr2* (**Figure 5C**). *Ccr1* and *Ccr2* have been known to be related to cell migration and chemotaxis. Considering the fact that peripheral blood immune cells need to cross blood vessels to play a role in tissues, we analyzed the differential expression of known genes related to cell migration. As shown in **Figure 5D** and **Figure 5E**, the expression of *Ccr1*, *Ccr2*, *Xcr1* and *Cxcr2* was up-regulated in the stress group as compared to the control group, suggesting that they played an important role in the migration of immune cells to tissues with emergency under acute stress.

### Acute stress leads to excessive lung inflammation in LPS-treated mice

The above bioinformatics analysis showed that acute stress could affect the gene expression profile of peripheral blood cells. Among these cells, myeloid cells other than lymphoid cells seemed to be ready to migrate from blood vessels to tissues to participate in inflammation. We tested this hypothesis. As shown in **Figure 6A**, LPS was injected intraperitoneally into mice to simulate microbial infection. 48 hours later, a lot of neutrophils and monocytes infiltrated into the lungs, but this phenomenon was not found in mice with simple acute stress. If LPS stimulation was performed after restrain stress, only neutrophil infiltration into the lung was further enhanced, while T cells and B cells showed a trend of decreased infiltration. We also performed H&E staining on lung tissue to assess the severity of pneumonia. The results showed that lungs of mice with LPS stimulation were characterized by inflammatory cell infiltration into alveolar interstitium, thickened alveolar walls and fluid exudation into alveoli. Such pathological changes were not found in the control group and stress group. Surprisingly, acute stress followed by LPS stimulation caused more serious pneumonia (**Figure 6B**). If mice received daily LPS stimulation, they would die occasionally. If LPS stimulation was performed after stress, the mortality of mice began to increase significantly after 4 days. The hazard ratio of mouse mortality under stress was 8.6 (**Figure 6C**). These data showed that acute stress led to an inflammatory state characterized by neutrophil mediated reaction, and repeated acute stress was harmful to the health of mice.

## Discussion

As we all know, chronic or long-term stress has many adverse effects on health (4, 5, 26). Acute or short-term stress could improve mobility and responsiveness for battle or flight, so as to promote the survival of life (14). Both acute and chronic stress affect the occurrence, progression, and outcome of diseases through the neuroendocrine-immune axis (15, 27). Although chronic stress seems to be more closely related to disease, immune changes caused by acute stress are often the basis of biological effects caused by chronic stress. Exploring the regulation of acute stress on the immune system not only helps to deeply understand the initiation of stress response, but also helps to explain how chronic stress affects the progress of disease. Previous studies have reported that acute stress could enhance the body’s immune response (16, 28, 29), but the characteristics of the immune response have not been described in detail.

Most immune cells are transported to tissues and organs through the circulatory system after they mature from bone marrow or thymus. Therefore, detecting the changes of peripheral blood immune cells could better reflect the impact of acute stress on the immune system. To avoid the problem of insufficient information obtained by traditional detection methods, we sequenced RNA extracted from peripheral blood to obtain biological big data, and then performed bioinformatics analysis. We found that 6 hours of restraint stress was enough to change the gene expression profile of peripheral blood immune cells, and most of DEG displayed up-regulated expression. The gene terms enriched by GSEA and GSVA were mainly related to inflammation, defense response, inflammatory response to wounding, pro-inflammatory cytokine production and so on. The core of these data was that acute stress may trigger an inflammatory state. This opinion was supported by the increase of serum proinflammatory cytokines after acute stress. Our results are consistent with previous literature that IL-6 is the dominant cytokine induced by acute stress in mice in the circulation (30). Similarly, a meta-analysis of several studies also showed that IL-6, TNF-α, and IL-1-β secretion were increased in response to acute stress in human (29). Among the 10 hub genes screened from PPI network, *Ilb* encodes one important pro-inflammatory cytokine, which is crucial for the occurrence of inflammation (31). *Tlr2*, *Cd14*, *Nlrp3* and *Clec7a* are involved in the signal pathways mediated by pattern recognition receptors, thus participating in the recognition of pathogenic microorganisms (32–35). *Fn1* is a fibrinogen-encoding gene and *Vegfa* is a growth factor-encoding gene, they are involved in the wound healing (36). *Lgals3* is a gene encoding Galectin-3, which has been considered as a modifier of anti-microbial immunity (37). *Ly6g*-encoded Ly6G has been regarded as neutrophil-specific marker in mice (38), suggesting an important participant of neutrophils in acute stress-induced response. *Fcgr3* is a gene encoding a receptor that binds IgG with high affinity and participates in IgG-mediated biological activity (39). Our results supported the previous hypothesis that acute or short-term stress prepares the immune system to respond to possible challenges such as injury or infection caused by stressors (such as predators, or modern medical/surgical procedures) (28, 40).

We evaluated the gene terms enriched by GSEA and GSVA in three aspects including cell activation, differentiation and chemotaxis. Obviously, acute stress enhanced the activation, differentiation and migration of granulocytes, especially neutrophils. However, the activation and differentiation of B cells and T cells were weakened under acute stress. Our data suggested that granulocytes were more active in the response induced by acute stress. Correspondingly, acute stress led to a significant decrease of T cells and B cells and a significant increase of neutrophils in peripheral blood, which supported our hypothesis. This phenomenon did not depend on the gender of mice (data not shown). Even in humans, the changes of immune cell populations in peripheral blood caused by acute stress are similar to those in mice (41). We agree with the opinion that stress response is a non-specific response of the body to stressors. In addition, previous studies have shown that acute stress could trigger the redistribution of immune cells in the body, in which hormones played an important regulatory role (42, 43). Our results showed that some DEG were involved in the process of granulocyte differentiation, which suggested that modifying the development and differentiation of different cell populations may be one of the reasons for the changes of peripheral blood cells under acute stress. This conjecture was supported by the analysis of hematopoietic stem and progenitor cells in the bone marrow under acute stress. We found that granulocyte-macrophage progenitor cells (GMP) increased in the bone marrow during acute stress (**Supplementary Figure 2**). Since GMP is the progenitor of granulocytes, the increase in its number under acute stress may lead to the generation of more neutrophils, which are then released into the peripheral blood.

The terminal site where immune cells play a role are usually tissues and organs. After infection or injury, tissues will release chemokines, which quickly attract and recruit immune cells from adjacent blood vessels (44). Under acute stress, we found that the enriched gene terms involved in cell chemotaxis or migration were mainly related to granulocytes and monocytes but not T cells and B cells. This highly suggested that acute stress prepares innate immune cells in peripheral blood to infiltrate into tissues with emergency. For cell migration, chemokines released by infected or damaged tissues determine the destination of cell migration, while the expression of chemokine receptors determines which cell types need to migrate (45). Our data showed that the expression of chemokine receptor-encoding genes including *Ccr1*, *Ccr2*, *Cxcr2* and *Xcr1* in the stress group was significantly higher than that in the control group. *Ccr1*-encoded CCR1 and *Cxcr2*-encoded CXCR2 are mainly expressed in neutrophils, and *Ccr2*-encoded CCR2 is mainly expressed in monocytes in the blood (46–48). This suggested that acute stress will mobilize peripheral blood neutrophils and monocytes to migrate to infected or injured tissues. To test the hypothesis, it is a good choice to take the inflammatory model as an amplifier. It was well known that peritoneal injection of LPS could induced system inflammation. Considering that the effect of acute stress gradually attenuates with the removal of the stressor, we detected the pathological status and immune cell infiltration of lung tissue on day 2 after LPS administration. Not surprisingly, LPS treatment led to obvious inflammatory pathological changes in the lungs of mice, and myeloid cells rather than lymphocytes were mainly accumulated in the lungs. Our data was consistent with previous studies that lymphocytes obviously infiltrated into lung tissue on day 4 after LPS treatment, while neutrophils and monocytes migrated to the lung earlier than lymphocytes (49, 50). This reflected the different migration speeds of different immune cell populations to inflammatory tissues. The opinion was supported in our previous study on LPS-induced neuroinflammation (51). Interestingly, if mice with acute inflammation were under acute stress, only neutrophil infiltration into lung tissue was enhanced. So, it was obvious that innate immune cells dominated by neutrophils played a central role in the inflammatory state caused by acute stress.

Although bioinformatics analysis has many advantages, its results need to be verified by experiments. Through a series of analysis strategies, we gradually guided the analysis results to the immune cell populations. Therefore, our validations were not for some genes, but for certain cell populations and pathological states. The reason why we did not pay attention to T cells and B cells was that their numbers in the peripheral blood are decreasing. In fact, lymphocytes in multiple organs showed a decreasing trend, and many of them migrated to the bone marrow (**Supplementary Figure 3**).

In LPS-induced systemic inflammation, repeated acute stress and LPS injection increased the risk of death in mice, which was consistent with the understanding that chronic stress was usually harmful to health. But what is the significance of acute stress-aggravated pneumonia? We could explain that excessive inflammation may be an important factor in promoting the death of mice, or we think that enhanced inflammation is a powerful tool against infection. Under acute stress, LPS-induced pneumonia seemed to be dominated by innate immunity represented by neutrophils, while adaptive immunity represented by T cells and B cells was in a contracting state. Previous data have shown that chronic restraint stress promoted lymphocyte apoptosis (18) and even weakened autoimmune diseases such as EAE (42). These data were consistent with our findings that acute restraint stress led to the reduction of peripheral blood lymphocytes. So, chronic stress effects may be the continuation and accumulation of acute stress effects. However, in other stress models, chronic stress could aggravate autoimmune diseases such as inflammatory bowel disease (52, 53). The regulation of immune system by chronic stress and acute stress may be very different. So, there is a significant gap in our understanding of the transitional phase between acute and chronic stress, studies on repeated acute stress might help fill this gap (54).

Altogether, our data showed that acute stress led to rapid mobilization of the immune system, and the body presented an inflammatory state dominated by innate immune response represented by neutrophils.

## Data availability statement

The datasets presented in this study can be found in online repositories. The names of the repository/repositories and accession number(s) can be found in the article/**Supplementary Material**.

## Ethics statement

The animal study was reviewed and approved by the Laboratory Animal Care Committee of Shandong First Medical University & Shandong Academy of Medical Sciences.

## Author contributions

HH designed the experiments; HH and LK wrote the manuscript and WS revised the manuscript. LT and NC performed bioinformatics analysis. YZ, JH and YL made animal model of disease and extracted RNA from blood. SY and YL performed flow cytometric analysis. All authors contributed to the article and approved the submitted version.

## Funding

This work was supported by grants from the National Natural Science Foundation of China (81971553, 82271803, 81971512), Shandong Introduction and Education Program of Yong Innovative Talents (rcjf005), Shandong Provincial key research and development program (2019GSF108256) and Shandong medical and health science and technology development project (2016WS0594).

## Conflict of interest

The authors declare that the research was conducted in the absence of any commercial or financial relationships that could be construed as a potential conflict of interest.

## Publisher’s note

All claims expressed in this article are solely those of the authors and do not necessarily represent those of their affiliated organizations, or those of the publisher, the editors and the reviewers. Any product that may be evaluated in this article, or claim that may be made by its manufacturer, is not guaranteed or endorsed by the publisher.
