# Supplementary material for: Acute stress induces an inflammation dominated by innate immunity represented by neutrophils in mice
Source: Front Immunol. 2022 Sep 29;13:1014296. doi: 10.3389/fimmu.2022.1014296 (PMC9556762; doi:10.3389/fimmu.2022.1014296)
Supplement: Supplementary file 2 [file DataSheet_1.docx]

Supplementary Figures

**Supplementary Figure 1.** Flow cytometry gating strategy for identifying immune cell populations.

**Supplementary Figure 2.** Analysis hematopoietic stem and progenitor cells under acute stress. After 6 hours of restraint stress (n=4) or no stress (n=4), bone marrow from mice were harvested for flow cytometric analysis. (A) Flow cytometry gating strategy for hematopoietic stem and progenitor cells. (B) Cell counts of granulocyte-macrophage progenitor (GMP), monocyte-DC progenitors (MDP) and common lymphoid progenitor (CLP) in the bone marrow. *P < 0.05, **P < 0.01. Means ± SD are shown. Data shown are representative of at least 2 independent experiments.

**Supplementary Figure 3.** Lymphocytes migrate to the bone marrow under acute stress. (A) After 6 hours of restraint stress (n=4) or no stress (n=4), single cell suspensions were acquired from spleen, liver, lung, lamina propria of small intestine (SI LP), lamina propria of large intestine (LI LP), thymus and bone marrow, followed by flow cytometric analysis. (B) Wild type CD45.2^+^ C57BL/6 mice received splenocytes (2.7×10^7^) from CD45.1^+^ congenic strain followed by restraint stress or no stress via tail vein. Six hours later, flow cytometry was used to detect the transferred cells in the bone marrow. Experimental design and flow cytometry gating strategy are shown. (C) The migration of adoptively transferred T cells and B cells to bone marrow in the stress group and the control group. *P < 0.05, **P < 0.01, ***P < 0.001, ****P < 0.0001. Means ± SD are shown. Data shown are representative of at least 2 independent experiments.
